# Downregulation of miR156-Targeted *PvSPL6* in Switchgrass Delays Flowering and Increases Biomass Yield

**DOI:** 10.3389/fpls.2022.834431

**Published:** 2022-02-18

**Authors:** Jinjun Cai, Wenwen Liu, Weiqian Li, Lijuan Zhao, Gang Chen, Yangyang Bai, Dongmei Ma, Chunxiang Fu, Yamei Wang, Xinchang Zhang

**Affiliations:** ^1^College of Natural Resources and Environment, Northwest Agriculture and Forestry University, Yangling, China; ^2^Institute of Agricultural Resources and Environment, Ningxia Academy of Agriculture and Forestry Sciences, Yinchuan, China; ^3^Shandong Provincial Key Laboratory of Energy Genetics and CAS Key Laboratory of Biofuels, Qingdao Institute of Bioenergy and Bioprocess Technology, Chinese Academy of Sciences, Qingdao, China; ^4^Breeding Base for State Key Laboratory of Land Degradation and Ecological Restoration in Northwest China, Ningxia University, Yinchuan, China; ^5^CAS Key Laboratory of Tibetan Medicine Research, Northwest Institute of Plateau Biology, Xining, China

**Keywords:** *PvSPL6*, flowering, biomass yield, subcellular localization, gibberellin, switchgrass

## Abstract

MiR156/*SQUAMOSA PROMOTER BINDING-LIKE*s (*SPL*s) module is the key regulatory hub of juvenile-to-adult phase transition as a critical flowering regulator. In this study, a miR156-targeted *PvSPL6* was identified and characterized in switchgrass (*Panicum virgatum* L.), a dual-purpose fodder and biofuel crop. Overexpression of *PvSPL6* in switchgrass promoted flowering and reduced internode length, internode number, and plant height, whereas downregulation of *PvSPL6* delayed flowering and increased internode length, internode number, and plant height. Protein subcellular localization analysis revealed that PvSPL6 localizes to both the plasma membrane and nucleus. We produced transgenic switchgrass plants that overexpressed a *PvSPL6*-*GFP* fusion gene, and callus were induced from inflorescences of selected PvSPL6-GFP_OE_ transgenic lines. We found that the PvSPL6-GFP fusion protein accumulated mainly in the nucleus in callus and was present in both the plasma membrane and nucleus in regenerating callus. However, during subsequent development, the signal of the PvSPL6-GFP fusion protein was detected only in the nucleus in the roots and leaves of plantlets. In addition, PvSPL6 protein was rapidly transported from the nucleus to the plasma membrane after exogenous GA_3_ application, and returned from the plasma membrane to nucleus after treated with the GA_3_ inhibitor (paclobutrazol). Taken together, our results demonstrate that *PvSPL6* is not only an important target that can be used to develop improved cultivars of forage and biofuel crops that show delayed flowering and high biomass yields, but also has the potential to regulate plant regeneration in response to GA_3_.

## Introduction

Flowering is the key physiological juncture at which the plant transitions from vegetative to reproductive growth, and flowering at the optimum time is important for plant growth and reproductive success. The control of flowering time is also critical for yield formation in cereal crops and biomass accumulation in biofuel crops (Wang and Ge, 2006). Switchgrass (*Panicum virgatum* L.) has been developed into a dedicated, herbaceous bioenergy crop (Fu et al., 2011), and biomass yield is a major target trait for the genetic improvement of switchgrass. The biomass yield of gramineous plants shows little increase after the transformation from vegetative to reproductive growth, as the nutrient supply flows primarily toward the inflorescence at this time (Casler, 2012). Therefore, the genetic manipulation of flowering time is a key approach for improving the architecture and biomass yield of switchgrass and other biofuel and forage crops (Wang and Ge, 2006; Johnson et al., 2017).

The molecular regulation of flowering time involves complex, synergistic regulation by exogenous and endogenous factors (Wang, 2014; Park et al., 2016; Campos-Rivero et al., 2017; Cho et al., 2017; Inoescu et al., 2017). In addition, mechanisms of flowering time regulation vary greatly among different plant species (Hill and Li, 2016). The regulatory mechanism of flowering time control in the model plant *Arabidopsis thaliana* has been studied extensively. It involves five major pathways: the photoperiodic, gibberellin, autonomous, vernalization, and age pathways. Among these five pathways, aging has received widely attention (Amasino and Michaels, 2010; Wang, 2014). The age pathway ensures that plants flower even under noninductive conditions (Poethig, 2009; Wang, 2014). MiR156 and its target *SQUAMOSA PROMOTER BINDING-LIKE* (*SPL*) genes constitute the key regulatory hub of the age pathway (Fu et al., 2012; Teotia and Tang, 2015). *SPL* genes encode a class of plant-specific transcription factors (TFs) and are conserved in monocots and eudicots (Klein et al., 1996; Yang et al., 2007). In *Arabidopsis*, *SPL* genes can be divided into three functionally distinct groups: (1) *SPL2*/*9*/*10*/*11*/*13*/*15* participate in developmental stage transition, *SPL9* and *SPL15* play a major role in these processes; (2) *SPL3*/*4*/*5* promote the floral meristem identity transition; and (3) *SPL6* does not have a major function in shoot morphogenesis, but may be important for certain physiological processes (Xu et al., 2016). *SPL* genes have been found to promote flowering mainly through three pathways: (1) SPL3/4/5 redundantly promote flowering through direct activation of *LEAFY* (*LFY*), *FRUITFULL* (*FUL*), and *AP1* (*LEAFY*; Yamaguchi et al., 2009); (2) SPL9 positively regulates the floral promoters *FUL*, *SUPPRESSOR OF OVEREXPRESSION OF CONSTANS1* (*SOC1*), and *AGL-LIKE 42* (*AGL42*; Wang et al., 2009). SPL9 can also promote the transcription of downstream miR172 (Wu et al., 2009), thereby inhibiting the expression of *APETALA2*-*LIKE* genes (*TARGET OF EAT1*, *TARGET OF EAT2*, *SCHLAFMUTZE*, and *SCHNARCHZAPFEN*; Aukerman and Sakai, 2003; Jung et al., 2007). The *AP2*-*like* genes can inhibit the expression of the flowering induction gene *FLOWERING LOCUS T* (*FT*). *FT* is induced by the photoperiodic pathway and regulated by *FUL* and *SOC1* under long-day conditions (Litt and Irish, 2003; Mathieu et al., 2007); and (3) SPL2/10/11, which have close homology to SPL9, can affect the flowering process by regulating *FUL* gene expression (Wang et al., 2009). In contrast to the extensive studies in *Arabidopsis*, little information is available on the flowering-related roles of *SPL*s in the Gramineae. The miR156-targeted *PvSPL*s in switchgrass belong to five orthologous groups (OGs): OG1, 2, 4, 9, and 10 (Wu et al., 2016). OG2 clade genes have the potential to participate in the regulation of reproductive development. *PvSPL6*, *PvSPL7′*, *PvSPL8*, and *PvSPL17* all belong to the OG2 clade. According to the latest research, PvSPL7 and PvSPL8 redundantly regulate flowering in switchgrass. Overexpression of the individual *SPL7* and *SPL8* gene promotes flowering, whereas their individual downregulation moderately delays flowering. Only simultaneous downregulation of *SPL7* and *SPL8* causes significant delayed flowering. PvSPL7 and 8 induce phase transition and flowering in grasses by directly upregulating *SEPALLATA3* (*SEP3*) and *MADS32* (Gou et al., 2019).

Recent studies have shown that the SPLs act as a key hub, integrating various flowering regulation pathways in *Arabidopsis* (Hong and Jackson, 2015; Teotia and Tang, 2015). Photoperiodic and gibberellin pathways have marked effect on the expression of some *SPL* genes. For photoperiodic pathway, the expression of *SPL3*/*4*/*5* is influenced by photoperiod in early vegetative stages (Jung et al., 2012). *FT* as the key component of the systemic flowering signal interacts with *FLOWERING LOCUS D* (*FD*), a meristem-specific bZIP transcription factor, in the shoot apex. FD binds directly to the G-box motifs present in the promoters of *SPL3*/*4*/*5*. Moreover, with respect to the changes in photoperiod, SOC1 binds to the CArG motifs present in the promoters of *SPL3*/*4*/*5* to regulate their expressions. Thus, photoperiod induction can induce SPL gene expression in a *CO*-, *SOC1*-, or *FT*-dependent manner (Teotia and Tang, 2015). For gibberellin pathway, GAs are a group of diterpenoid phytohormones that regulate a variety of events in plant development, including seed germination, stem elongation, leaf expansion, flowering, and fruit development (Sun, 2010; Mcatee et al., 2013; Tuan et al., 2018; Bao et al., 2020). GAs have been shown to regulate these diverse biological processes mainly by overcoming the inhibition of the DELLA proteins, a family of nuclear repressors of the GA response. Because DELLA proteins do not contain canonical DNA-binding domains, they regulate downstream genes by interacting with other TFs (Daviere et al., 2008; Hauvermale et al., 2012; Locascio et al., 2013; Xu et al., 2014). Growing evidence indicates that GA signaling and the miR156/*SPL*s module are connected through direct interactions between DELLAs and SPL TFs. For example, the GA-induced flowering pathway can be integrated into the miR156-mediated flowering pathway through interactions between DELLAs and SPLs. The binding of DELLAs to SPLs has been shown to impair the transcriptional activation of downstream SPL target genes. Consequently, DELLAs delay the floral transition by reducing SPL15-mediated expression of *MADS*-*box* genes (*SOC1* and *FUL*) in the shoot apex or by repressing the activation of *FT* through inhibition of SPL9 in the leaves (Galvão et al., 2012; Yu et al., 2012; Hyun et al., 2016). In addition, recent studies have shown that the DELLA-SPL9 module is involved in axillary bud formation. SPL9 inhibits the transcription of the axillary bud identity gene *LAS*, while binding of DELLA to SPL9 attenuates the repression of *LAS* by SPL9, thereby promoting axillary bud initiation (Zhang et al., 2020). However, given the fact that TFs are usually expressed in a tissue-specific and temporally variable manner, questions remain about the contribution of *SPL*s to GA signaling at the tissue or single-cell level. Systematic protein–protein interaction assays and visualization of protein-TF interactions *in vivo* will help us to address this question.

In the past two decades, the proteolysis of membrane-bound TFs (MTFs) has been studied extensively as a novel transcriptional regulatory mechanism (Chen et al., 2008; Seo et al., 2008). MTFs are TFs with transmembrane domains (TMs) that are fixed to membranes in a dormant state. When exposed to developmental and environmental cues, some MTFs undergo proteolytic cleavage, releasing intracellular fragments into the nucleus to control gene transcription (Liu et al., 2008; Seo et al., 2010a,b). As a result, MTFs can rapidly respond to pressures from extracellular or intracellular stimuli (Hoppe et al., 2000). MTFs have been observed in many types of organisms, including plants, animals, and microorganisms (Hoppe et al., 2001; Yang et al., 2014; Xie et al., 2015). Consistent with the activation pathways of more typical TFs, these molecules are delicately regulated at many points throughout the signal transduction process. Cellular stimuli can activate MTF precursors and induce their translocation. Cellular translocation signals include ligand-receptor binding response signals, growth hormones, and many types of stress, including temperature, drought, and salinity (Popovic et al., 2007; Seo et al., 2010a,b; Ma et al., 2012; De Clercq et al., 2013; Misra et al., 2013). Signal transduction in response to stress can enable the visualization of protein–TF interactions. In plants, studies of MTFs have focused on two major TF families, NAM/ATAF1/2/CUC2 (NAC) and basic leucine zipper (bZIP; Chen et al., 2008; Seo et al., 2008). To date, eight *NAC*, three *bZIP*, one *MYB*, and one *PHD* TF have been identified and characterized (Seo, 2014). However, other TF families that contain MTFs have not previously been reported.

In this work, we demonstrate that *PvSPL6*, a miR156-targeted member of the *SPL* family, can regulate flowering time in switchgrass. As the number of same orthologous group with PvSPL7 and PvSPL8, PvSPL6 can independently regulate the flowering time. Inhibition of *PvSPL6* expression causes a markedly delays in flowering. Besides, unlike the homolog AtSPL3/4/5 in *Arabidopsis*, PvSPL6 protein has both nuclear and plasma membranes localization. However, the dual localization of nuclear and plasma membranes only appears in the regeneration stage during switchgrass development process. Exogenous GA_3_ application induces the rapid nucleus to plasma membrane translocation of PvSPL6 proteins, and the GA_3_ inhibitor (paclobutrazol) application induces the plasma membrane returned to nucleus translocation of PvSPL6 proteins. Hence, *PvSPL6* may be an excellent candidate for genetic modification and improvement of biomass production in bioenergy crops. Furthermore, it is possible to discover a new function and mechanism of PvSPL6 in regulating regeneration by studying how PvSPL6 localization responds to GA pathway.

## Materials and Methods

### Plant Materials and Growth Conditions

The wild-type control and transgenic switchgrass plants were generated from a high-quality embryogenic callus line with a single genotype that was obtained by screening a mass of Alamo switchgrass (*P. virgatum* L.) seed. The Alamo switchgrass seed was derived from Noble Research Institute, Ardmore, United States. Embryogenic callus of wild-type control and transgenic plants was cultured in a sterile culture room at 23°C with a 16 h light/8 h dark photoperiod (390 μE/m^2^/s) and 80% relative humidity. Wild-type control and transgenic plants were planted in a greenhouse at 26°C under a 16 h light/8 h dark photoperiod (390 μE/m^2^/s) and approximately 60% relative humidity. The development of switchgrass plants was divided into five elongation stages (E1–E5) and three reproductive stages (R1–R3) as described previously (Moore et al., 1991; Hardin et al., 2013).

### Vector Construction and Plant Transformation

The predicted cDNA sequence of *PvSPL6* (*Pavir.2KG430400*) from the switchgrass genome database v4.1^1^ was used to design primers for cloning the full-length coding region and RNAi fragment of *PvSPL6*. About 275 bp fragment representing a non-conserved region in the 5′-UTR and coding sequence of *PvSPL6* was selected as the RNAi fragment. This design can rule out the offtarget effect on other miR156-targeted *SPL* genes. The amplified PCR products were confirmed by Sanger sequencing, respectively. For the overexpression of *PvSPL6*, the correct full-length coding region was inserted into the binary pANIC6B vector by LR recombination reactions (Invitrogen, United States). The pANIC6B vectors contain the *attR1*-*Cm^r^*-*ccdB*-*attR2* cassette for overexpression of the target gene, a plant selectable marker cassette (*hygromycin phosphotransferase*, *hph*), and a visual reporter gene cassette (*GUSPlus*; Mann et al., 2012). For suppression of *PvSPL6*, the verified RNAi fragment was cloned into the RNAi-mediated suppression vector pANIC8B driven by the maize *Ubiquitin* promoter (Mann et al., 2012). The main difference between pANIC6B and pANIC8B is that the pANIC8B vectors contain the *attR1*-*Cm*^r^-*ccdB*-*attR2* cassette downstream of an inverted repeat of itself, resulting in a hairpin loop of the target sequence after recombination and transcription. Then the constructed vectors were transferred into *Agrobacterium tumefaciens* strain EHA105 using the freeze–thaw method (Chen et al., 1994). To generate transgenic plants, the embryogenic callus line with a single genotype was employed for *Agrobacterium*-mediated transformation following the procedure described previously (Xi et al., 2009). The control switchgrass plants were generated by using empty pANIC6B and pANIC8B empty vectors, respectively.

For the construction of vector to observe PvSPL6 subcellular localization, the *GFP* was fused to the C-terminal of *PvSPL6* coding region, and then inserted into the pANIC6B vector by LR recombination reactions. Then verified constructs were transferred into EHA105 and introduced into the embryogenic callus line by *Agrobacterium*-mediated transformation. Hygromycin (Phytotechlab, Lenexa, United States) was used as the selection reagent for the production of PvSPL6_OE_, PvSPL6_RNAi_, and PvSPL6-GFP_OE_ transgenic switchgrass plants. Positive transgenic lines were identified by PCR using specific *hph*, *PvSPL6*, and *PvSPL6*-RNAi primers (Supplementary Table 1). The expected sizes of the PCR products were 375, 642, and 275 bp, respectively.

### Subcellular Localization Assay

*PvSPL6* cDNA fragments encoding the N-terminal membrane spanning domain (amino acid 1–71, cDNA 1–213 bp, *PvSPL6*-N) and the remainder (amino acid 72–214, cDNA 214–642 bp, *PvSPL6*-C) were amplified by PCR, respectively. The full-length and two truncated coding sequences of *PvSPL6* were fused with *GFP* and ligated into the pCambia1300 vector. EHA105 containing the final binary vector pCambia1300::*PvSPL6*-*GFP*, pCambia1300::*PvSPL6*-N-*GFP*, or pCambia1300::*PvSPL6*-C-*GFP* was injected into leaves of four-week old *Nicotiana benthamiana*. P19 from tomato bushy stunt virus was used to inhibit transgenic silencing (Chen et al., 2007). The resulting fluorescence signal was observed 48–72 h after injection using a FluoView FV1000 confocal laser scanning microscope (Olympus, Japan). The fluorescent dye propidium iodide (PI) was used as a cell plasma membrane marker, the 4′,6-diamidino-2-phenylindole (DAPI) was used as a cell nuclear marker. The primer pairs used for vector construction are listed in Supplementary Table 1.

### Quantitative Real-Time PCR Analysis

Total RNA was extracted from switchgrass stems using a TRIzol kit (TransGen Biotech, Beijing, China) and was reverse-transcribed into cDNA using a PrimeScript RT Reagent Kit with gDNA Eraser (Takara, Dalian, China) according to the manufacturer’s instructions. Quantitative real-time PCR (qRT-PCR) was performed in a 20-μl reaction volume that contained 10 μl of SYBR Premix ExTaq (Takara, Dalian, China), 2 μl of cDNA (first strand cDNA, diluted five times), and 0.5 μM of each primer. The primer pairs used for qRT-PCR are listed in Supplementary Table 1. *PvUBQ2* (*Pavir.1KG065600*) was used as the reference for normalization (Huang et al., 2014). The cycle thresholds were determined using a Roche Light Cycler 480 II sequence detection system (Roche, Shanghai, China).

### Phenotypic Measurements

Flowering time, internode number, internode length, tiller number, and plant height were measured on three biological replicates when plants reached the R1 stage. The I2 internodes were used for the measurement of internode length.

### Confocal Laser Scanning Microscopy After Hormone and Plant Growth Regulator

Embryogenic callus were induced from inflorescences of selected three PvSPL6-GFP_OE_ lines. These callus was cultured on SM5 medium {MS0 + 5 mg/L 2,4-D (2,4-Dichlorophenoxyacetic acid) + 0.15 mg/L 6-BA [N-(Phenylmethyl)-9H-purin-6-amine]} supplemented with different hormones and plant growth regulators for 2 weeks. For hormones and growth regulators treatments, different concentrations of 2,4-D (0, 1, 3, and 5 mg/L), 6-BA (0.02, 0.05, 0.1, and 1 mg/L), 6-Furfurylamino-purine (KT; 0, 0.5, 1, and 4 mg/L), gibberellin (GA_3_; 0, 10, 100, and 400 mg/L), and paclobutrazol (0, 0.5, 1, and 2 mg/L) were used, respectively. The fluorescence signal of each callus type under each hormone and growth regulator treatment was observed 48–72 h later using a FluoView FV1000 confocal laser scanning microscope (Olympus, Japan).

### Statistical Analysis

Three control switchgrass plants, three PvSPL6_OE_ lines, and three PvSPL6_RNAi_ lines were statistical analyzed in this work. The selected transgenic plants were propagated simultaneously with three biological replicates. One-way ANOVA was used for qRT-PCR and phenotypic statistical analysis, and treatment means were separated using Duncan’s multiple range test (*p* < 0.05). All the statistical analyses were performed with the SPSS software (IBM SPSS Statistics 25.0, United States).

## Results

### Molecular Cloning and Sequence Analyses of Switchgrass SBP Transcription Factor *PvSPL6*

**Figure 1:**
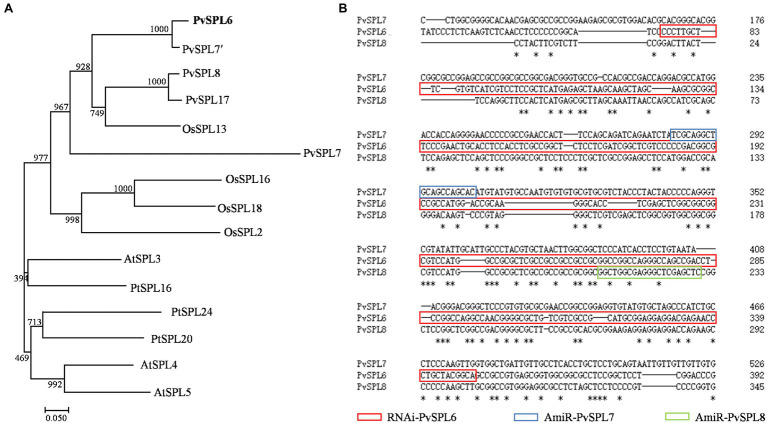
Molecular characterization and sequence analysis of *PvSPL6*. **(A)** Phylogenetic analysis of *SQUAMOSA PROMOTER BINDING-LIKEs* (SPLs) in clade OG2 in dicot and monocot plant species. A maximum likelihood tree was constructed in PhyML version 3.0 based on multiple alignments of deduced protein sequences from *Panicum virgatum* (PvSPL6, Pavir.2KG430400; PvSPL7′, Pavir.2NG503700; PvSPL8, Pavir.2KG430000; and PvSPL17, Pavir.2NG503500), *Oryza sativa* (OsSPL2, LOC_Os01g69830; OsSPL13, LOC_Os07g32170; OsSPL16, LOC_Os08g41940; and OsSPL18, LOC_Os09g32944), *Arabidopsis thaliana* (AtSPL3, AT2G33810; AtSPL4, AT1G53160; and AtSPL5, AT3G15270), and *Populus trichocarpa* (PtSPL16, Potri.011G055900; PtSPL20, Potri.001G398200; and PtSPL24, Potri.007G138800). Bootstrap support values (>50%) based on 1,000 replications are given at the nodes. The sequence data were retrieved from Phytozome and/or Genbank. **(B)** Sequence alignment of *PvSPL6*, *PvSPL7*, and *PvSPL8*. The red box indicates the RNAi regions of *PvSPL6*; the blue box indicates the artificial microRNA (amiRNA) regions of *PvSPL7*; and the green box indicates the amiRNA regions of *PvSPL8*. The artificial microRNA regions mean the nonconserved regions of *PvSPL7* and *8*. The amiRNAs of *PvSPL7* and *8* were to knockdown the expression levels of *PvSPL7* and *8*, respectively. *means the identical nucleotide.

Blastn searches against the switchgrass genome (*P. virgatum* v4.1, Phytozome) indicated that these four OG2 genes, *PvSPL6* (*Pavir.2KG430400*), *PvSPL7′* (*Pavir.2NG503700*), *PvSPL8* (*Pavir.2KG430000*), and *PvSPL17* (*Pavir.2NG503500*), were located on chromosome 2. *PvSPL6* and *PvSPL7′* as an allele share over 86% sequence identity, and *PvSPL8* and *PvSPL17* as an allele share 90.7% sequence identity (Supplementary Figure 1). Another OG2 gene, *PvSPL7*, has also been reported recently (Gou et al., 2019). We used orthologs of the five PvSPLs in OG2 (PvSPL6, PvSPL7′, PvSPL8, PvSPL17, and PvSPL7) from three genome-sequenced species (*A. thaliana*, *Populus trichocarpa*, and *Oryza sativa*) to construct a phylogenetic tree. The tree showed that PvSPL6 and PvSPL7′ clustered together in a group, implying that they have a close evolutionary relationship and similar functions. By contrast, the distance between PvSPL6 and PvSPL7 on the phylogenetic tree suggested that they may have different functions (Figure 1A). The sequence alignment further revealed the variation among *PvSPL6*, *PvSPL7*, and *PvSPL8* as well (Figure 1B). These results prompted us to explore PvSPL6 in more detail. Using information from the assembled switchgrass genome database at Phytozome, the full-length sequence of *PvSPL6* was isolated to study its function in switchgrass.

### The PvSPL6 TF Shows Both Nuclear and Plasma Membrane Localization

**Figure 2:**
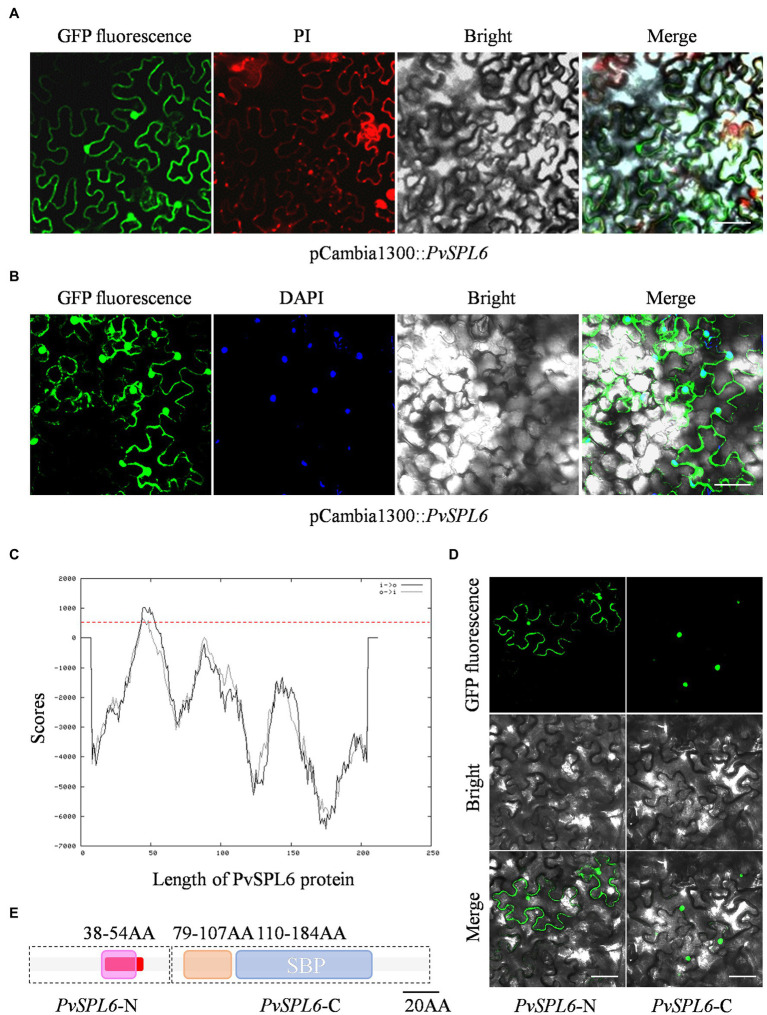
Subcellular localization and bioinformatics analysis of PvSPL6. **(A)** and **(B)** Subcellular localization assays of PvSPL6. *Agrobacterium* cells harboring fusion constructs were infiltrated into the abaxial surfaces of *Nicotiana benthamiana* leaves, and samples were observed 72 h later under a GloMax 20/20 single tube luminometer (Promega, United States). GFP fluorescence, green fluorescent signal; PI, propidium iodide signal; DAPI, 4′,6-diamidino-2-phenylindole signal; Bright, bright field signal; and Merge, superimposed signal. Scale bar = 20 μm. **(C)** Transmembrane domain prediction of PvSPL6 by TMPred. The red dotted line indicates a score of 500 (scores above 500 are considered significant). Black solid line means inside to outside helices; black dotted line means outside to inside helices. **(D)** Subcellular localization assays of PvSPL6-N and PvSPL6-C. GFP fluorescence, green fluorescent signal; Bright, bright field signal; and Merge, superimposed signal. Scale bar = 20 μm. **(E)** Functional domain predictions of PvSPL6. The boxes indicate functional domains. Purple box means the first functional domain with low compositional complexity; orange box means the second functional domain with low compositional complexity; and blue box means conserved SBP domain. The red section indicates the predicted transmembrane region.

A fused vector containing *PvSPL6* and *GFP* was constructed to investigate the subcellular localization of *PvSPL6*. After Sanger sequencing validation, the pCambia1300::*PvSPL6*-*GFP* vector was introduced into tobacco leaves by infiltration with *A. tumefaciens* strain EHA105 to produce transient expression. Unlike miR156-targeted PvSPL2 and PvSPL4, the PvSPL6-GFP signal was located in both the nucleus and the plasma membrane (Figures 2A,B; Supplementary Figure 2A). We then used TMPred^2^ to predict TMs in PvSPL6. The results showed that PvSPL6 had one potential transmembrane helix from amino acids 41 to 58 (red label), with a score of 1,035 (only scores above 500 are considered significant; Figure 2C). PvSPL2 and PvSPL4 did not contain predicted transmembrane helices (Supplementary Figure 2B), consistent with the results of the subcellular localization analysis. To determine the authenticity of TMPred predict, two truncated coding sequences of *PvSPL6*, *PvSPL6*-N (contains the N-terminal membrane spanning domain) and *PvSPL6*-C (remainder) were fused with *GFP* and ligated into the pCambia1300 vector. Transient expression in tobacco leaves showed that the PvSPL6-C-GFP signal was located entirely in the tobacco cell nucleus, whereas the PvSPL6-N-GFP signal was located in both the nucleus and plasma membrane (Figure 2D). The SMART tool^3^ was used for functional domain analysis. PvSPL6 was shown to have three functional domains, located at amino acids 38–54, amino acids 79–107, and amino acids 110–184, respectively (Figure 2E). Among them, the functional domain located at amino acids 110–184 is squamosa promoter binding protein (SBP) domain. It is a highly conserved domain of *SPL* transcription factor family consisting of approximately 78 amino acid residues. Moreover, the coding sequences of the functional domain located at amino acids 38–54 of PvSPL6 was highly coincident with the N-terminal membrane spanning domain. Hence, this functional domain of PvSPL6 has the potential to determine its membrane localization.

### Morphological Characterization of *PvSPL6* Transgenic Switchgrass Plants

**Figure 3:**
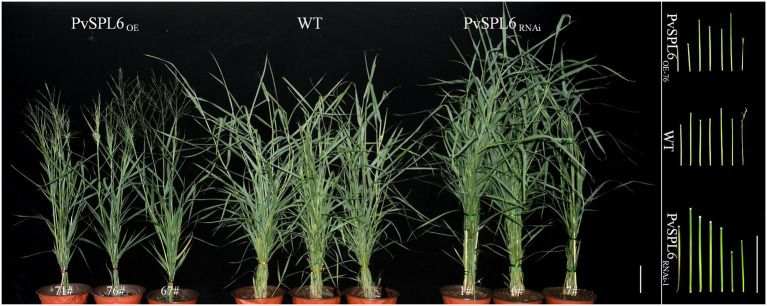
Morphological characterization of *PvSPL6* transgenic switchgrass plants. Morphological characterization of PvSPL6_OE_ and PvSPL6_RNAi_ transgenic switchgrass plants. Scale bar = 10 cm.

**Figure 4:**
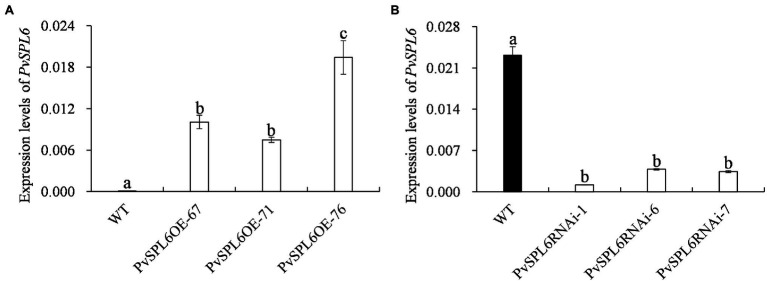
Quantitative real-time PCR (qRT-PCR) analysis of *PvSPL6* transcript levels in different PvSPL6_OE_ and PvSPL6_RNAi_ transgenic switchgrass plants. **(A)** qRT-PCR analysis of *PvSPL6* transcript levels in different PvSPL6_OE_ transgenic switchgrass plants. **(B)** Quantitative real-time PCR analysis of *PvSPL6* transcript levels in different PvSPL6_RNAi_ transgenic switchgrass plants. *PvUBQ2* was used as a reference for normalization. The values are the means ± SEs (*n* = 3). The letters above the error bars indicate significant differences determined by one-way ANOVA (*p* < 0.05, Duncan’s multiple-range test).

**Figure 5:**
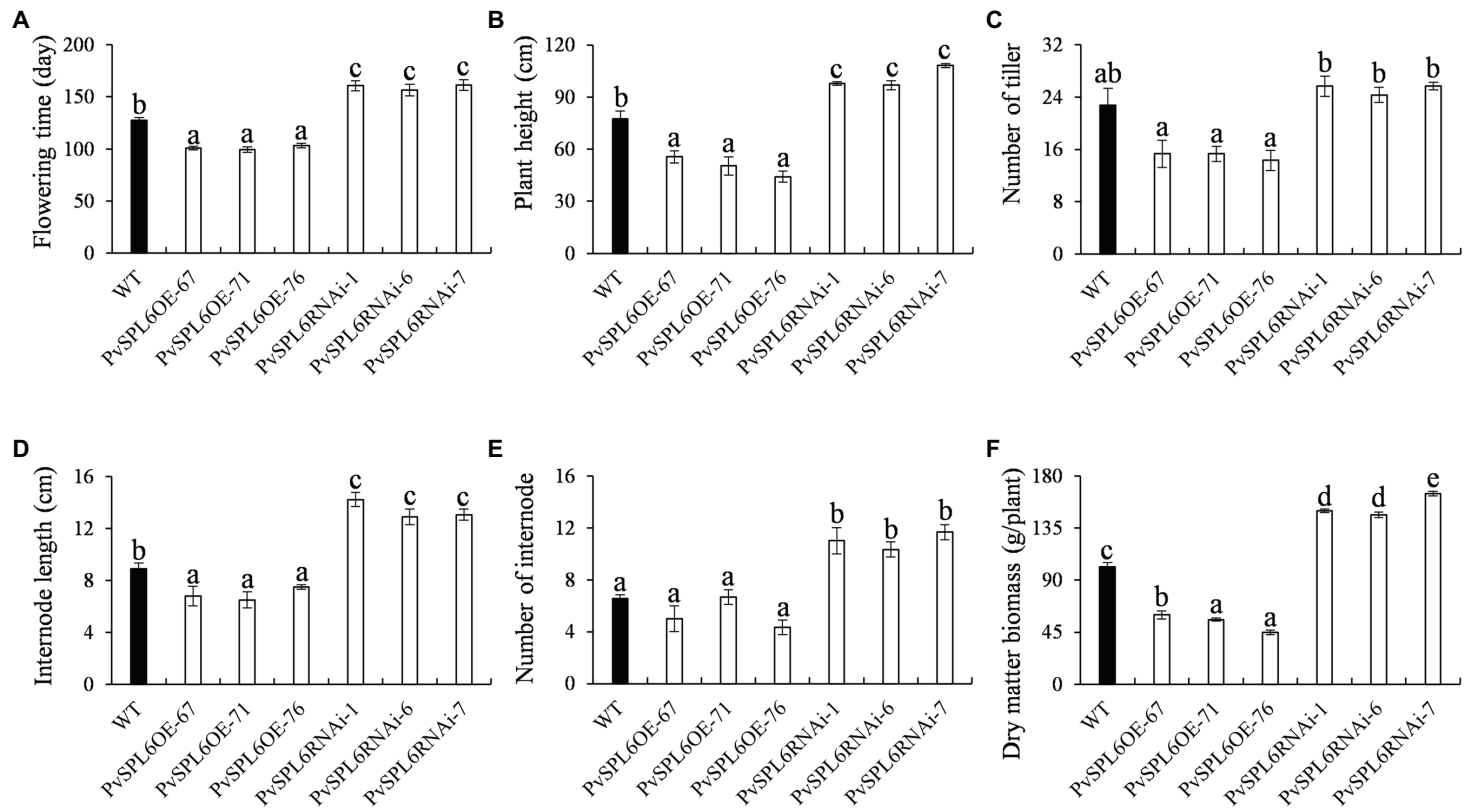
Phenotypic statistics of PvSPL6_OE_ and PvSPL6_RNAi_ transgenic switchgrass plants. Comparisons of **(A)** flowering time, **(B)** plant height, **(C)** number of tiller, **(D)** internode length, **(E)** number of internode, and **(F)** dry matter biomass. The values are the means ± SEs (*n* = 3). The letters above the error bars indicate significant differences determined by one-way ANOVA (*p* < 0.05, Duncan’s multiple-range test).

To characterize the function of *PvSPL6* in switchgrass, we constructed *PvSPL6* overexpression and RNAi vectors and introduced them into wild-type switchgrass callus by *Agrobacterium*-mediated transformation (Supplementary Figure 3). Compared with the wild-type control, the PvSPL6_OE_ lines consistently displayed markedly earlier heading dates, reduced internode lengths and numbers, and shorter plant heights. By contrast, the PvSPL6_RNAi_ lines showed conspicuously delayed heading dates and increased internode lengths and numbers (Figure 3). We selected the three PvSPL6_OE_ lines with the highest expression levels (PvSPL6_OE-67_, _-71_, and _-76_) and the three PvSPL6_RNAi_ lines with the lowest expression levels (PvSPL6_RNAi-1_, _-6_, and _-7_) for further phenotypic analysis (Figure 4; Supplementary Figure 4). Taken together, our results showed that *PvSPL6* overexpression and suppression altered plant development. Upregulation of *PvSPL6* shortened the vegetative growth period and decreased dry biomass yield by 40.90, 44.96, and 55.80% in the three lines. Downregulation of *PvSPL6* extended the vegetative growth period and increased the dry biomass yield by 47.73, 44.50, and 62.54% (Figure 5). To exclude the possibility that other genes in the same clade were inhibited by *PvSPL6* RNAi, we also measured the expression levels of *PvSPL7′*, *PvSPL7*, *PvSPL8*, and *PvSPL17* in PvSPL6_RNAi_ transgenic plants. Only *PvSPL6* expression was inhibited in PvSPL6_RNAi_ plants relative to the wild-type, confirming that the phenotype of the PvSPL6_RNAi_ plants was caused by reduced expression of *PvSPL6* alone (Figure 4B; Supplementary Figure 5). The phenotypes of the two transgenic plant types indicated that *PvSPL6* functions in the control the flowering time and affects internode elongation in switchgrass. To explain the observed phenotype, we roughly validated how PvSPL6 participates in regulation of floral transitions. The high correlation between the expression levels of *PvSPL6* and *PvSEP3*/*PvMADS32*, the target genes of PvSPL7 and PvSPL8, in different transgenic lines suggested that PvSPL6 has the similar regulatory mechanism in floral transitions to PvSPL7/8 (Supplementary Figure 6).

### Subcellular Localization of PvSPL6 in Different Tissues

**Figure 6:**
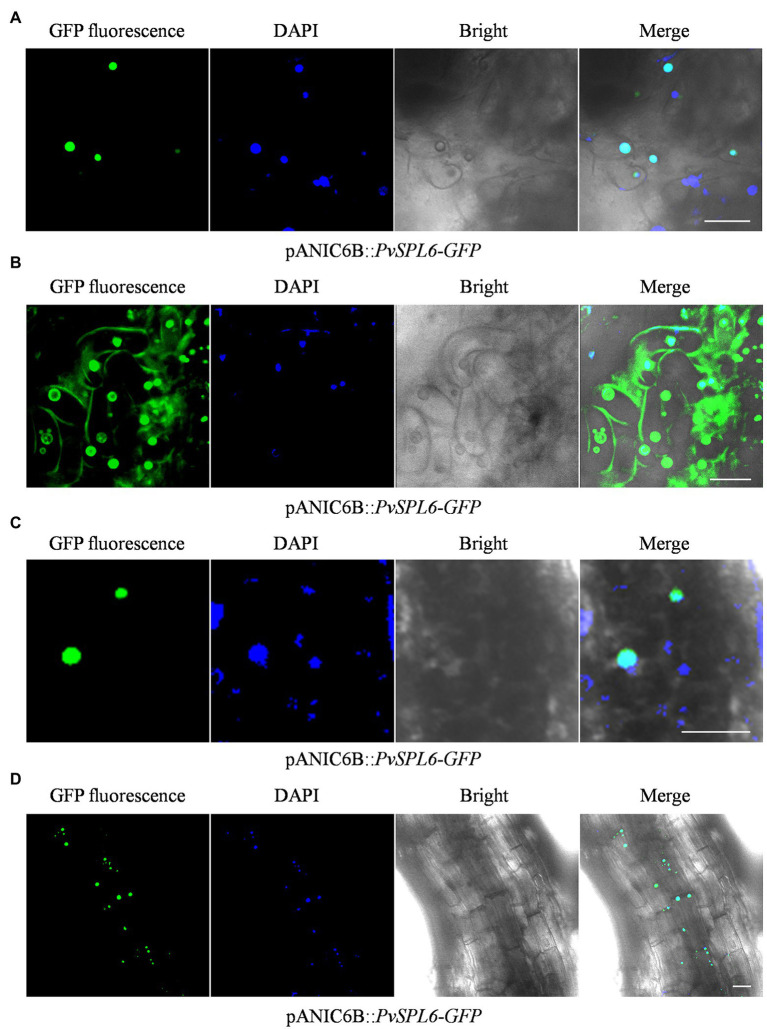
Subcellular localization analysis of different tissues in PvSPL6-GFP_OE_ transgenic plants. **(A)** Subcellular localization analysis of undifferentiated embryogenic callus of PvSPL6-GFP_OE_ transgenic plants. **(B)** Subcellular localization analysis of differentiated embryogenic callus of PvSPL6-GFP_OE_ transgenic plants. **(C)** Subcellular localization analysis of leaves from PvSPL6-GFP_OE_ transgenic plants. **(D)** Subcellular localization analysis of roots from PvSPL6-GFP_OE_ transgenic plants. GFP fluorescence, green fluorescent signal; DAPI, 4′,6-diamidino-2-phenylindole signal; Bright, bright field signal; and Merge, superimposed signal. Scale bar = 20 μm.

To further study the localization of PvSPL6, the verified pANIC6B::*PvSPL6*-*GFP* constructs were transferred into a high-quality embryogenic callus line with a single genotype. By *Agrobacterium*-mediated transformation, we produced PvSPL6-GFP_OE_ transgenic plants. The PvSPL6-GFP_OE_ transgenic plants had phenotypes similar to those of the PvSPL6_OE_ plants. We induced embryogenic callus from three PvSPL6-GFP_OE_ transgenic lines, and we observed PvSPL6-GFP signal only in the nuclei of the undifferentiated transgenic callus (loose and irregular and have not yet formed somatic embryos; Figure 6A). This was not consistent with the results, we observed in tobacco leaf cells. However, we observed partial translocation of the PvSPL6-GFP signal from the nucleus to the plasma membrane when the callus was in the differentiation stage (compact and dense somatic embryo, and even appear green bud points; Figure 6B). During subsequent development, the differentiated calli formed complete switchgrass plants. Confocal laser scanning microscopy showed that PvSPL6 exhibited its complete nuclear localization in both leaves and roots of the resulting plants (Figures 6C,D).

### GA_3_ Controls the Localization of PvSPL6 in Switchgrass

**Figure 7:**
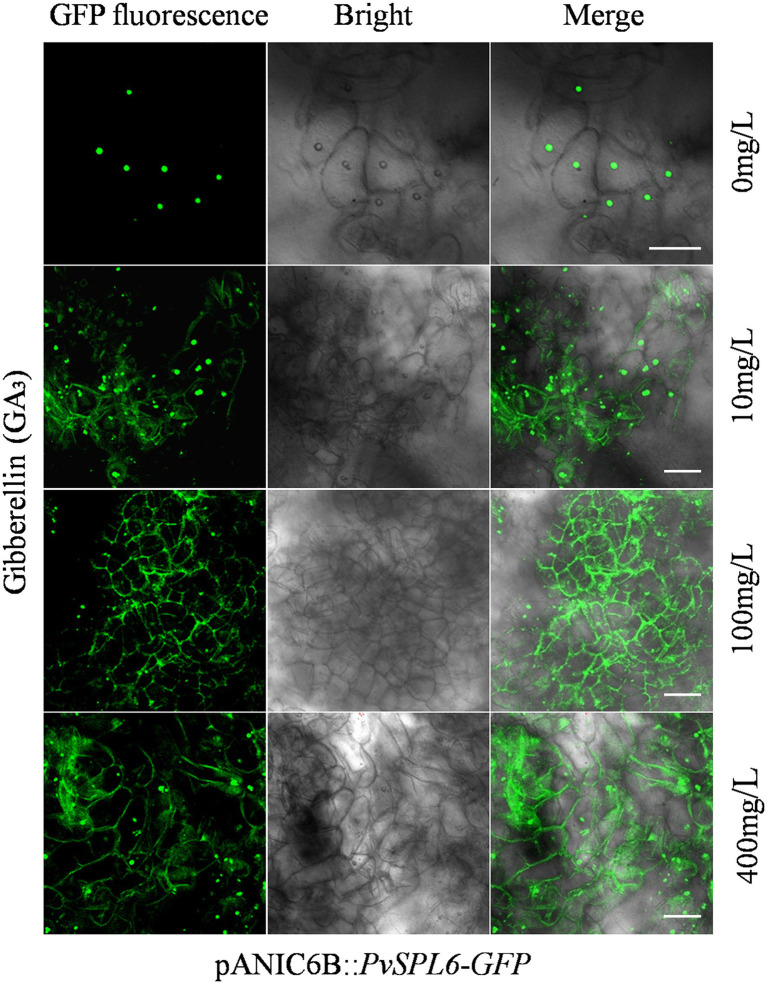
Subcellular localization analysis of embryogenic callus from PvSPL6-GFP_OE_ transgenic plants in response to GA_3_. Subcellular localization of PvSPL6 in response to different concentrations of GA_3_ (0, 10, 100, and 400 mg/L). GFP fluorescence, green fluorescent signal; Bright, bright field signal; and Merge, superimposed signal. Scale bar = 20 μm.

To investigate the biological significance of PvSPL6 membrane localization, we first needed to identify the factor and related pathway to which PvSPL6 localization responds. Ligand-receptor binding response signals, growth hormones, and many types of stress may be the candidates for influencing PvSPL6 localization. Combined the flowering phenotype of *PvSPL6* transgenic plants and the roles of GA pathway in flowering regulation, we chose GA_3_ to treat PvSPL6-GFP_OE_ callus. Predictably, we observed partial PvSPL6-GFP signal translocation from the nucleus to the plasma membrane when *PvSPL6* transgenic callus was treated with different concentrations of GA_3_ (0, 10, 100 and 400 mg/L). Compared with callus in SM5 medium without GA_3_, callus treated with even a low concentration of GA_3_ (10 mg/L) showed clear plasma membrane localization of PvSPL6-GFP. This plasma membrane localization became more obvious as the GA_3_ concentration increased (Figure 7). We also used various other plant growth regulators treatments, 2,4-D (0, 1, 3, and 5 mg/L), 6-BA (0.02, 0.05, 0.1, and 1 mg/L), and KT (0, 0.5, 1, and 4 mg/L), to assess the hormone specificity of the PvSPL6 response. Embryogenic callus from three PvSPL6-GFP_OE_ lines was cultured on SM5 medium supplemented with the above compounds at 23°C in the dark for 2 weeks, and the subcellular localization of PvSPL6 was observed by confocal laser scanning microscopy. PvSPL6 maintained its nuclear localization after treatment with all concentrations of 2,4-D, 6-BA, and KT (Supplementary Figure 7). Furthermore, to further confirm the effect of GA_3_ on PvSPL6 localization, we cultured differentiated callus with dual localization of nuclear and plasma membrane in medium supplemented with different concentrations of GA_3_ inhibitors (paclobutrazol, 0, 0.5, 1, and 2 mg/L). The results showed that PvSPL6-GFP signal translocation from the plasma membrane returned to the nucleus in differentiated callus after paclobutrazol treatment. And this phenomenon became more obvious as the increase of concentration (Supplementary Figure 8). In conclusion, the localization of PvSPL6 is more sensitive to plant endogenous hormones compared with plant growth regulators. And GA_3_ is the crucial factor responsible for the plasma membrane localization of PvSPL6 in cells.

## Discussion

Precise flowering time is critical to reproductive success. Since the discovery that miR156, whose expression decreases with age, mediates the regulation of flowering time in plants, the miR156-*SPL*s module has attracted significant attention as the core regulatory hub of the age pathway. To date, the *SPL* family has been found to promote flowering mainly through three pathways in *Arabidopsis*. SPL3/4/5, SPL9, and SPL2/10/11 are dominant in each pathway, respectively. In contrast to the extensive studies in *Arabidopsis*, little information is available on the flowering-related roles of *SPL*s in the Gramineae. OG2 clade genes have the potential to participate in the regulation of reproductive development in switchgrass. *PvSPL6*, *PvSPL7′*, *PvSPL8*, *PvSPL17*, and *PvSPL7* all belong to the OG2 clade. Based on molecular characteristics and sequence analysis of genes, the subfamily was further divided into three branches: *PvSPL6* and *PvSPL7′*, *PvSPL8* and *PvSPL17*, and *PvSPL7*. Among them, *PvSPL6* and *PvSPL7′*, *PvSPL8* and *PvSPL17* as the allele showed high degree of sequence similarity and close evolutionary relationship. *PvSPL7* showed significant divergence from the *SPL* genes belongs to the same subfamily. Currently, Only *PvSPL7* and *8* have been functionally identified in switchgrass. Overexpression of *PvSPL7* and *8* promotes flowering, whereas downregulation of individual genes moderately delays flowering. Simultaneous downregulation of *PvSPL7* and *8* results in extremely delayed or nonflowering plants (Gou et al., 2019). We therefore studied the function of *PvSPL6* in the present study and found that *PvSPL6* regulated phase transition and flowering in switchgrass. Downregulation of *PvSPL6* by itself significantly delayed flowering, suggesting that *PvSPL6* may be the dominant gene in this subfamily for the regulation of flowering time in switchgrass.

Subcellular localization assays showed that PvSPL6 was localized to both the nucleus and the plasma membrane, unlike its SPL3/4/5 homologs in *Arabidopsis*. Transmembrane domain prediction showed that PvSPL6 contained a transmembrane structure that was not present in AtSPL3/4/5 or in other SPL subfamilies of switchgrass. As an MTF, *PvSPL6* may therefore have unique functions or mechanisms. Large-scale expression profiling of plant MTF genes and phenotypic analyses of available mutants show that MTFs are involved in diverse developmental processes and growth hormone signaling (Kim et al., 2007). The transcriptional control conferred by the activation of plant MTFs is thought to have a wide array of regulatory roles in diverse aspects of plant growth and development. Meanwhile, our data showed that the nuclear and plasma membrane dual localization of PvSPL6 only occurred at the stage of callus differentiation during the whole development process, so we speculated that the plasma membrane localization of PvSPL6 had the potential to participate in switchgrass regeneration. But these speculations need to be further verified. Thus, research on the functional implications of PvSPL6 plasma membrane localization may break new ground and provide additional clues for understanding the molecular mechanisms by which TF activity is regulated.

Furthermore, relevant studies have shown that MTFs mediate diverse aspects of stress response and enable the rapid regulation of transcription under stressful conditions. The rapid turnover of membrane-bound proteins is essential for cell survival, as is the maintenance of a minimum level of physiological activity under stress conditions (Vik and Rine, 2000; Poon and Jans, 2005). Our data indicated that PvSPL6 proteins were rapidly transported from the nucleus to the plasma membrane after exogenous GA_3_ application, and returned from the plasma membrane to nucleus after treated with the GA_3_ inhibitor (paclobutrazol). Moreover, PvSPL8, the same subfamily with PvSPL6, also showed both nuclear and plasma membrane localization, and responded to GA_3_ treatment (Supplementary Figure 9). These results confirm that the plasma membrane localization of PvSPL6 subfamily indeed respond to GA_3_ signal and have a directly or indirectly related to GA pathway. Previous studies have shown that the binding of DELLAs, components of GA signaling, to SPLs blocks the transcriptional activation of their downstream target genes. DELLAs delay the floral transition by reducing the SPL15-mediated expression of MADS-box genes (*SOC1* and *FUL*) in the shoot apex or by repressing the activation of *FT* in leaves by inhibiting SPL9 (Galvão et al., 2012; Yu et al., 2012; Hyun et al., 2016). In addition, SPL9 represses transcription of the axillary bud identity gene *LATERAL SUPPRESSOR* (*LAS*), and the binding of DELLA to SPL9 attenuates germination (Zhang et al., 2020). We therefore speculate that PvSPL6 may respond to a specific protein in the GA signaling pathway. After receiving this protein signal, the PvSPL6 TF may be activated and then translocated from the nucleus to the membrane, thus curtailing its TF activity in the nucleus. The biological processes and regulatory mechanisms associated with the transportation of PvSPL6 from the nucleus to the plasma membrane in switchgrass are still largely unknown, but it is worth investigating in the future.

## Data Availability Statement

The datasets presented in this study can be found in online repositories. The names of the repository/repositories and accession number(s) can be found in the article/Supplementary Material.

## Author Contributions

XZ, JC, YW, and CF designed the research. JC, YW, WenL, WeiL, LZ, GC, and YB performed the experiments. JC, YM, XZ, CF, and DM analyzed the data. XZ, JC, and YW wrote the article. All authors contributed to the article and approved the submitted version.

## Funding

This research was supported by the National Key Research and Development Project (2016YFC0501702), Special Projects for The Central Government to guide The Development of Local Science and Technology (2021FRD05023), Ningxia Hui Autonomous Region Key R&D Project (2020BCF01001), Biological Resources Programme, Chinese Academy of Sciences (KFJ-BRP-007-018), and Special Project of Ningxia Academy of Agriculture and Forestry Science and Technology Cooperation with Foreign (DW-X-2020002).

## Conflict of Interest

The authors declare that the research was conducted in the absence of any commercial or financial relationships that could be construed as a potential conflict of interest.

## Footnotes

^1^ https://phytozome-next.jgi.doe.gov/

^2^ https://embnet.vital-it.ch/

^3^ http://smart.embl-heidelberg.de/
