# Supplementary material for: Downregulation of miR156-Targeted PvSPL6 in Switchgrass Delays Flowering and Increases Biomass Yield
Source: Front Plant Sci. 2022 Feb 18;13:834431. doi: 10.3389/fpls.2022.834431 (PMC8894730; doi:10.3389/fpls.2022.834431)
Supplement: Supplementary file 1 [file Data_Sheet_1.docx]

**Supplementary Figure 1**


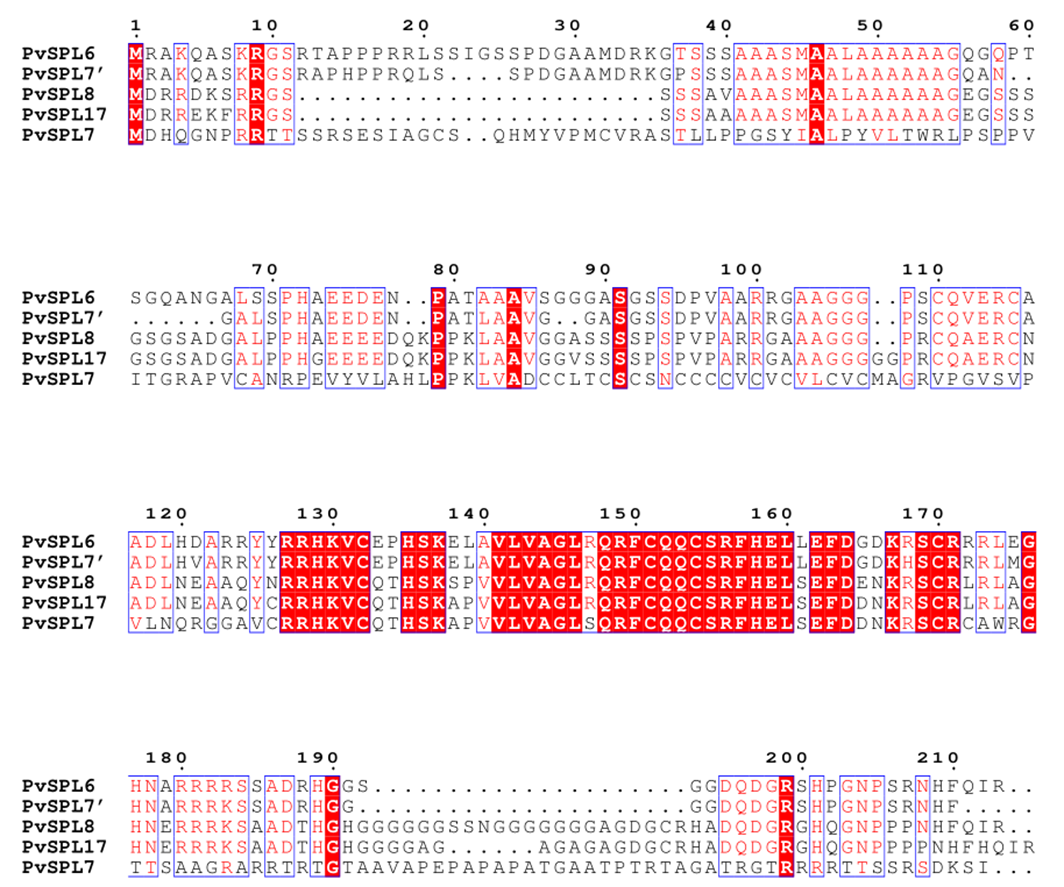


**Supplementary Figure 1.** **Comparative analysis of the amino acid sequences of PvSPL6, PvSPL7′, PvSPL8, PvSPL17, and PvSPL7.**

**Supplementary Figure 2**


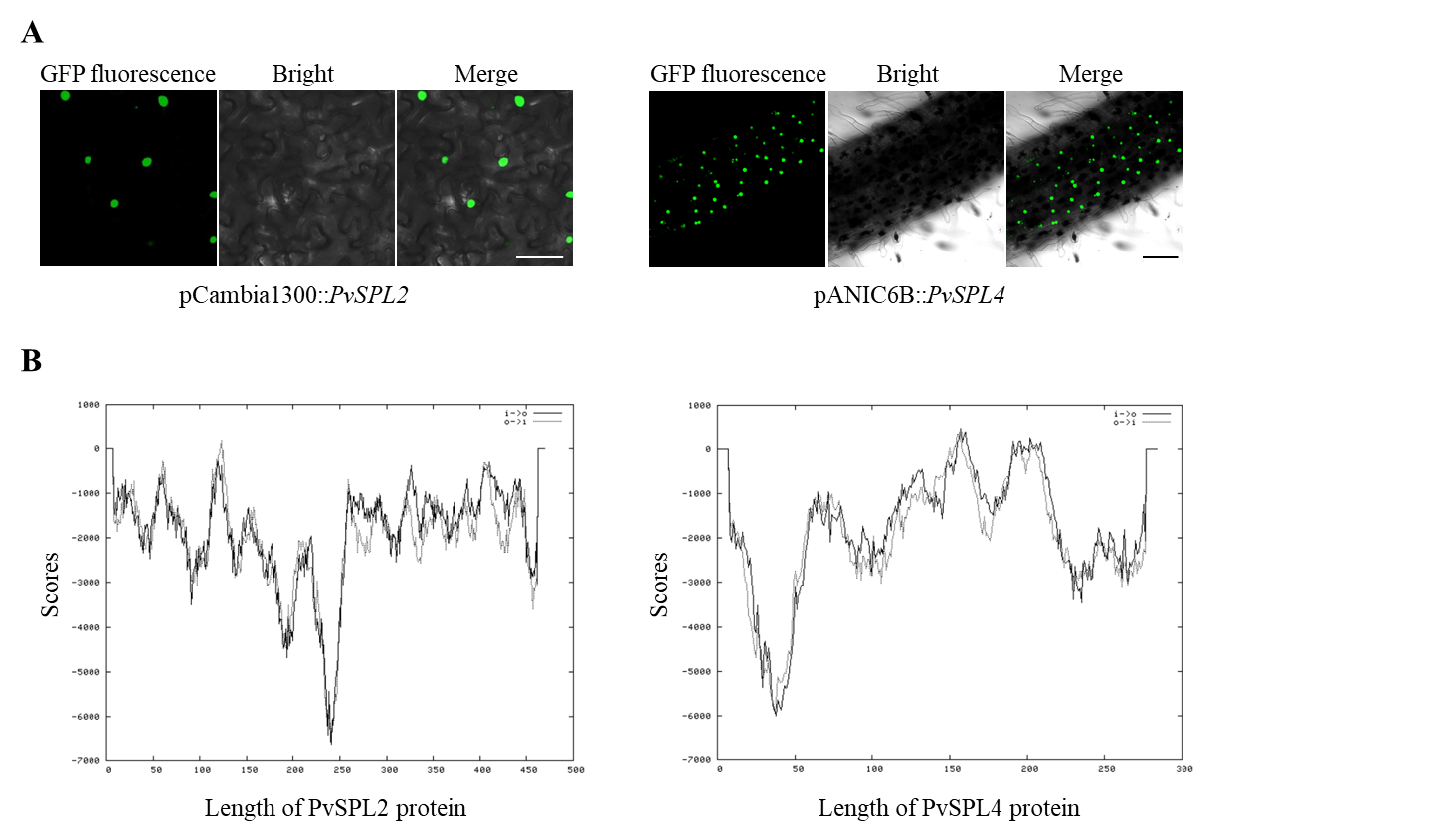


**Supplementary Figure 2.** **Subcellular localization and transmembrane domain analysis of PvSPL2 and PvSPL4.** **(A)** Subcellular localization assays of PvSPL2 and PvSPL4. *Agrobacterium* cells harboring the PvSPL2-GFP fusion constructs were infiltrated into the abaxial surface of *N*. *benthamiana* leaves, and the samples were observed 72 h later under a GloMax 20/20 single tube luminometer (Promega, USA); *Agrobacterium* cells harboring the PvSPL4-GFP fusion constructs were introduced into the wild-type switchgrass, and the samples were observed under a GloMax 20/20 single tube luminometer (Promega, USA). GFP fluorescence, green fluorescent signal; Bright, bright field signal; Merge, superimposed signal. Scale bar = 20 µm. **(B)** Transmembrane domain prediction of PvSPL2 and PvSPL4 by TMPred; the red dotted line indicates a score of 500, and scores above 500 are considered significant. Black solid line means inside to outside helices; black dotted line means outside to inside helices.

**Supplementary Figure 3**


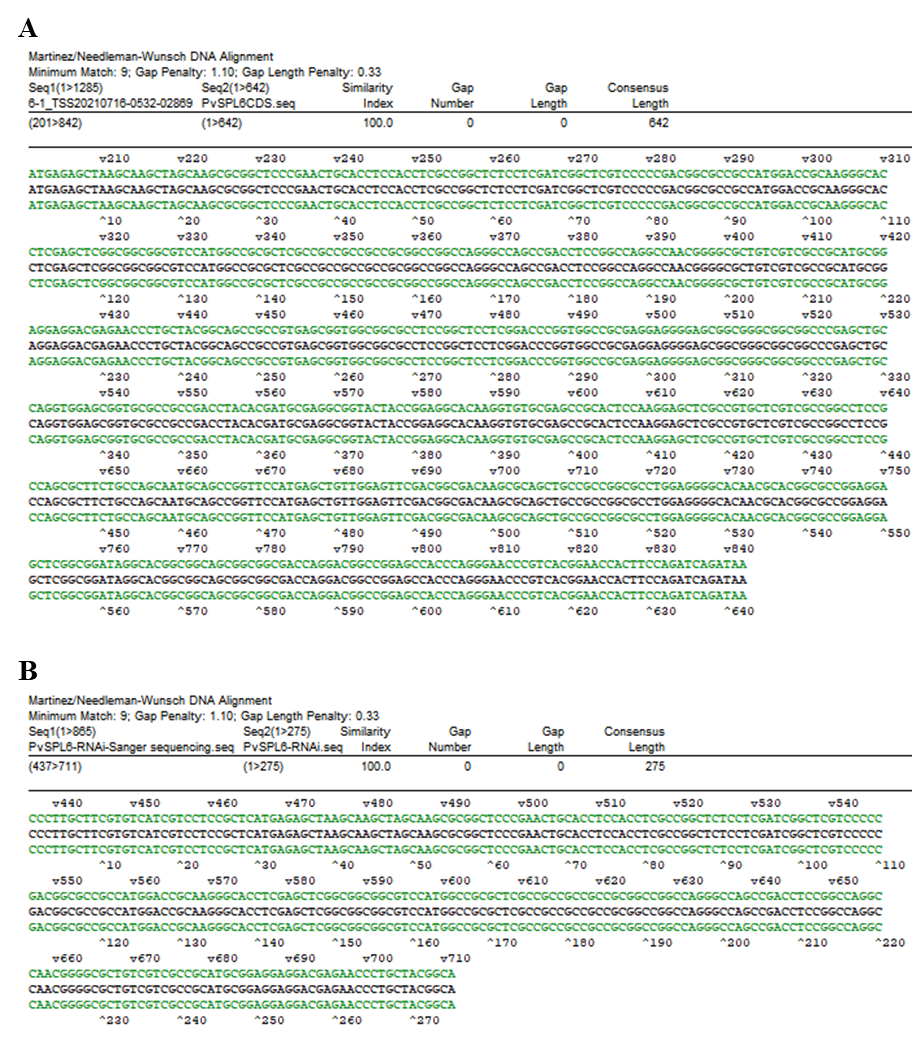


**Supplementary Figure 3. Alignment of *PvSPL6* and *PvSPL6*-RNAi sequences by Sanger sequencing. (A)** Alignment of *PvSPL6* sequence by Sanger sequencing. The first sequence is the cDNA sequence of *PvSPL6* by Sanger sequencing; the second sequence is the predicted cDNA sequence of *PvSPL6* from the switchgrass genome database; the third sequence is the sequence alignment similarity of sequence 1 and 2. **(B)** Alignment of *PvSPL6*-RNAi sequence by Sanger sequencing. The first sequence is the cDNA sequence of *PvSPL6*-RNAi by Sanger sequencing; the second sequence is the predicted cDNA sequence of *PvSPL6*-RNAi from the switchgrass genome database; the third sequence is the sequence alignment similarity of sequence 1 and 2.

**Supplementary Figure 4**


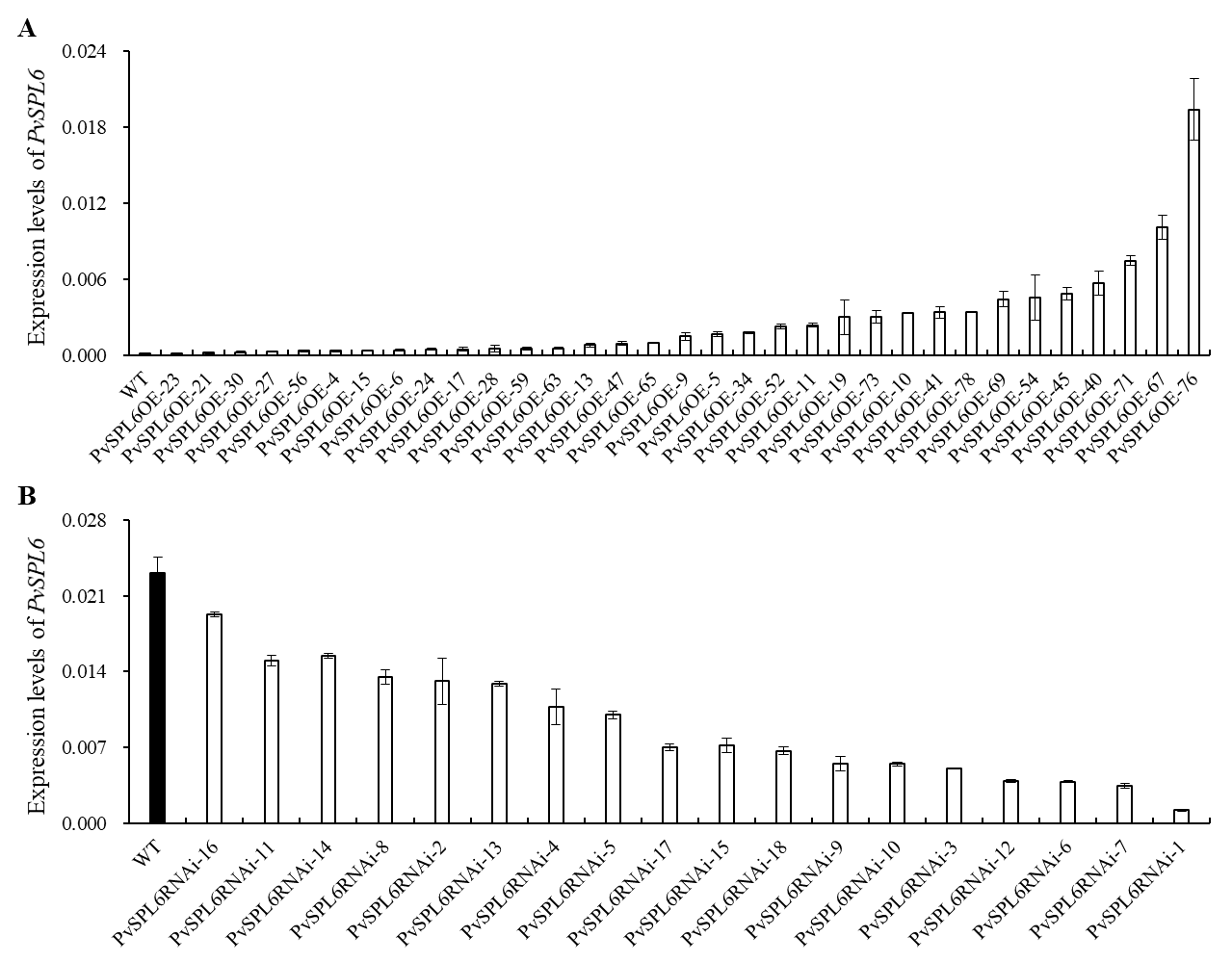


**Supplementary Figure 4. Quantitative real-time PCR analysis of *PvSPL6* transcript levels in different *PvSPL6* transgenic switchgrass plants. (A)** Quantitative real-time PCR analysis of *PvSPL6* transcript levels in PvSPL6_OE_ transgenic switchgrass plants. **(B)** Quantitative real-time PCR analysis of *PvSPL6* transcript levels in PvSPL6_RNAi_ transgenic switchgrass plants. *PvUBQ2* was used as the reference for normalization. The values are the means ± SEs (n=3).

**Supplementary Figure 5**


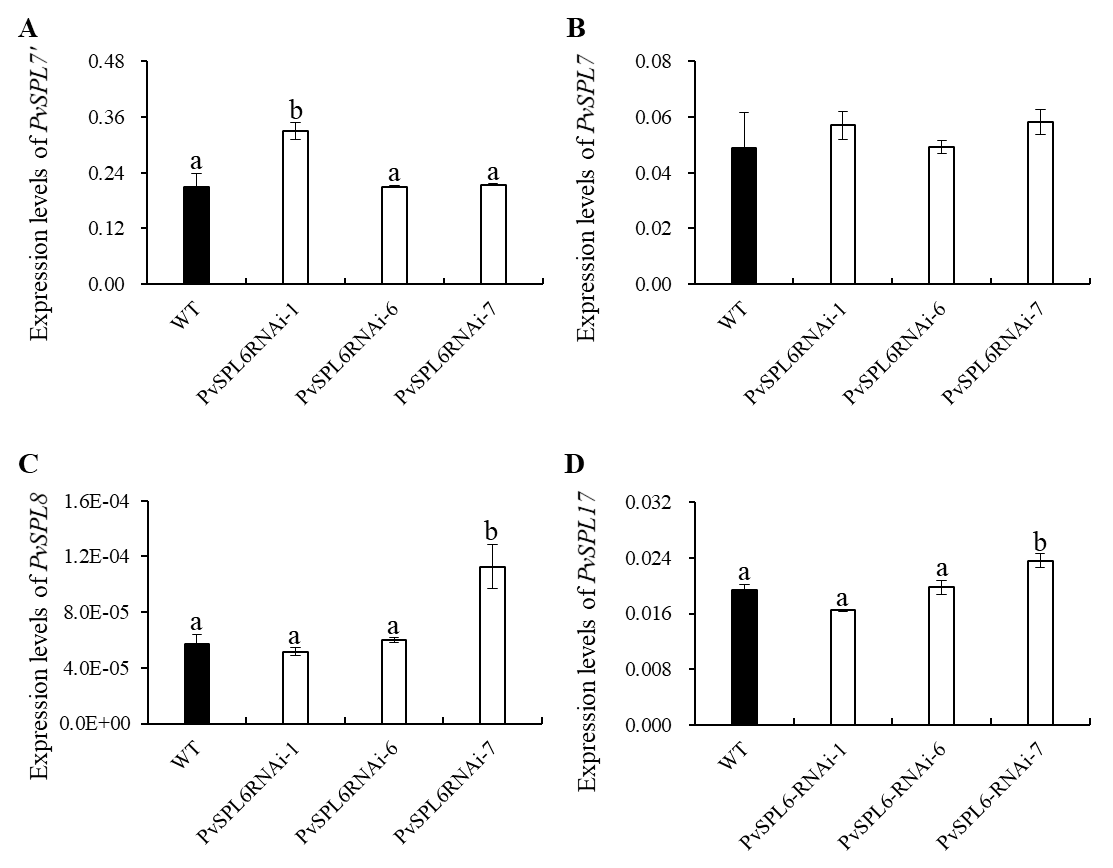


**Supplementary Figure 5.** **Quantitative real-time PCR** **analysis of *PvSPL7′*, *PvSPL7*,** ***PvSPL8*, and *PvSPL17* transcript levels in PvSPL6_RNAi_ transgenic switchgrass plants.** **(A)** Quantitative real-time PCR analysis of *PvSPL7′* transcript levels in PvSPL6_RNAi_ transgenic switchgrass plants. **(B)** Quantitative real-time PCR analysis of *PvSPL7* transcript levels in PvSPL6_RNAi_ transgenic switchgrass plants. **(C)** Quantitative real-time PCR analysis of *PvSPL8* transcript levels in PvSPL6_RNAi_ transgenic switchgrass plants. **(D)** Quantitative real-time PCR analysis of *PvSPL17* transcript levels in PvSPL6_RNAi_ transgenic switchgrass plants. *PvUBQ2* was used as a reference for normalization. The values are the means ± SEs (n=3). The letters above the error bars indicate significant differences determined by one-way ANOVA (p<0.05, Duncan’s multiple-range test).

**Supplementary Figure 6**


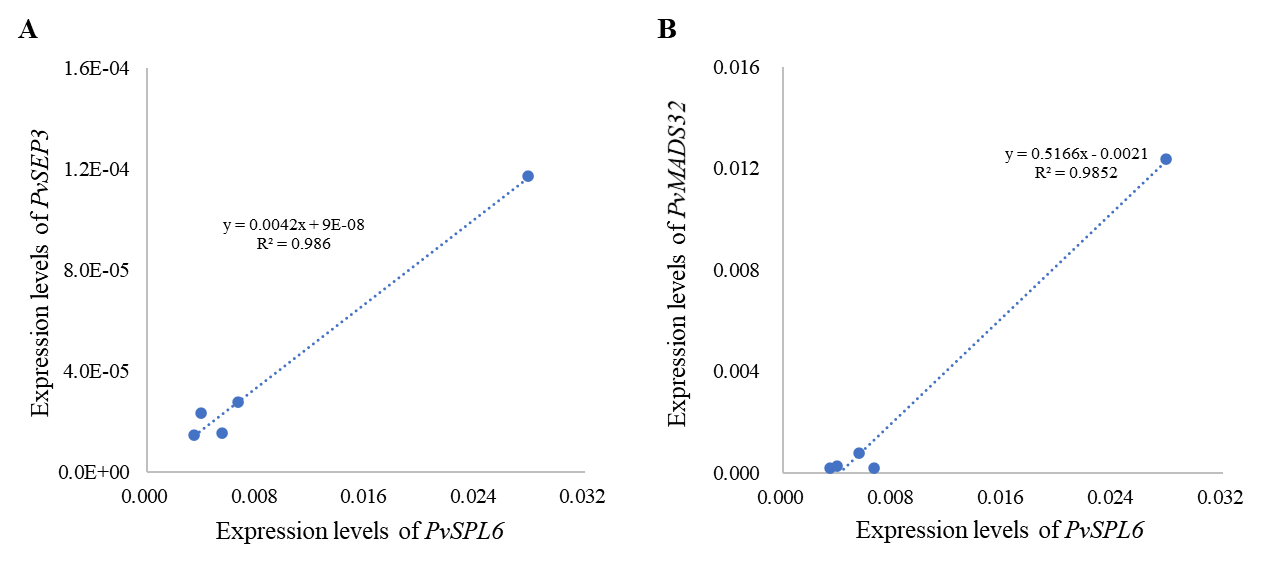


**Supplementary Figure 6. Quantitative real-time PCR analysis of *PvSPL6* and potential downstream genes transcript levels in different *PvSPL6* transgenic switchgrass plants. (A)** Relationship between expression levels of *PvSPL6* and *PvSEP3*. Blue dot indicates *PvSPL6* transgenic line. **(B)** Relationship between expression levels of *PvSPL6* and *PvMADS32*. Blue dot indicates *PvSPL6* transgenic line. *PvUBQ2* was used as the reference for normalization. The values are the means ± SEs (n=3).

**Supplementary Figure 7**


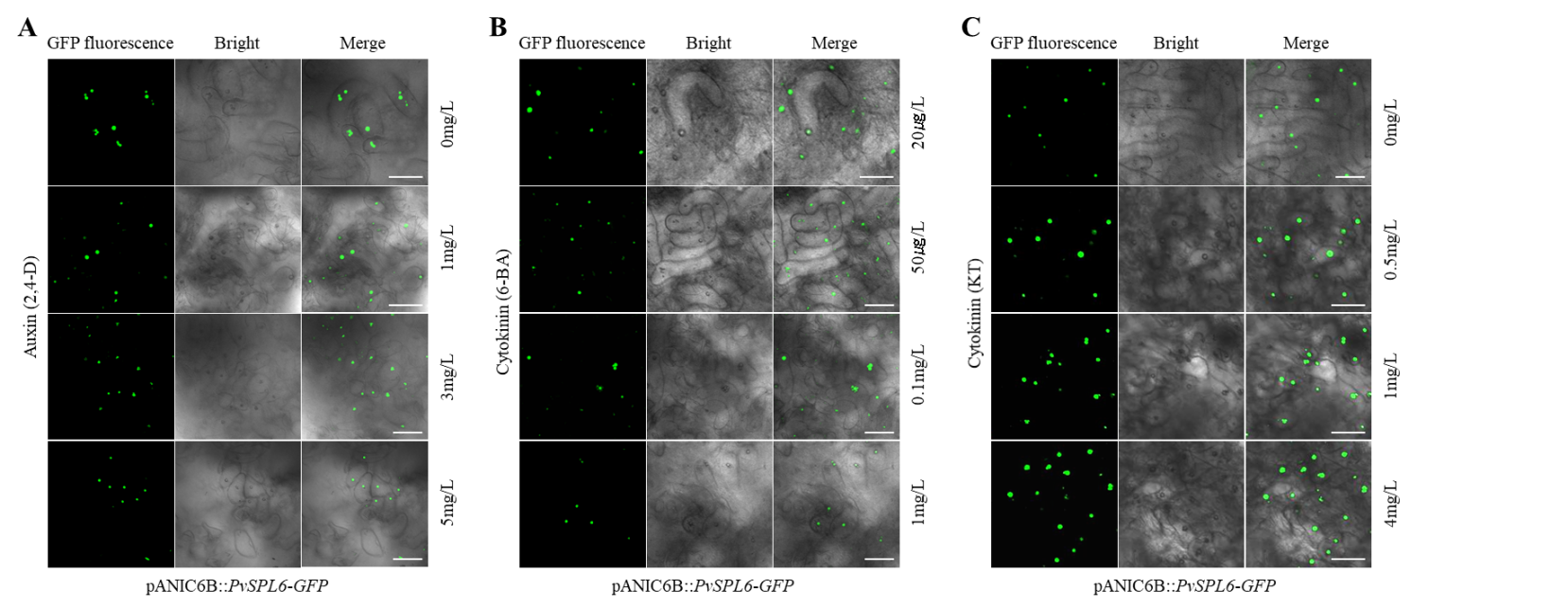


**Supplementary Figure 7.** **Subcellular localization analysis of embryogenic callus of PvSPL6-GFP_OE_ transgenic plants in response to 2,4-D, 6-BA, and KT.** **(A)** Subcellular localization of PvSPL6 in response to different concentrations of 2,4-D (0, 1, 3, and 5 mg/L). **(B)** Subcellular localization of PvSPL6 in response to different concentrations of 6-BA (0.02, 0.05, 0.1, and 1 mg/L). **(C)** Subcellular localization of PvSPL6 in response to different concentrations of KT (0, 0.5, 1, and 4 mg/L). GFP fluorescence, green fluorescent signal; Bright, bright field signal; Merge, superimposed signal. Scale bar = 20 µm.

**Supplementary Figure 8**


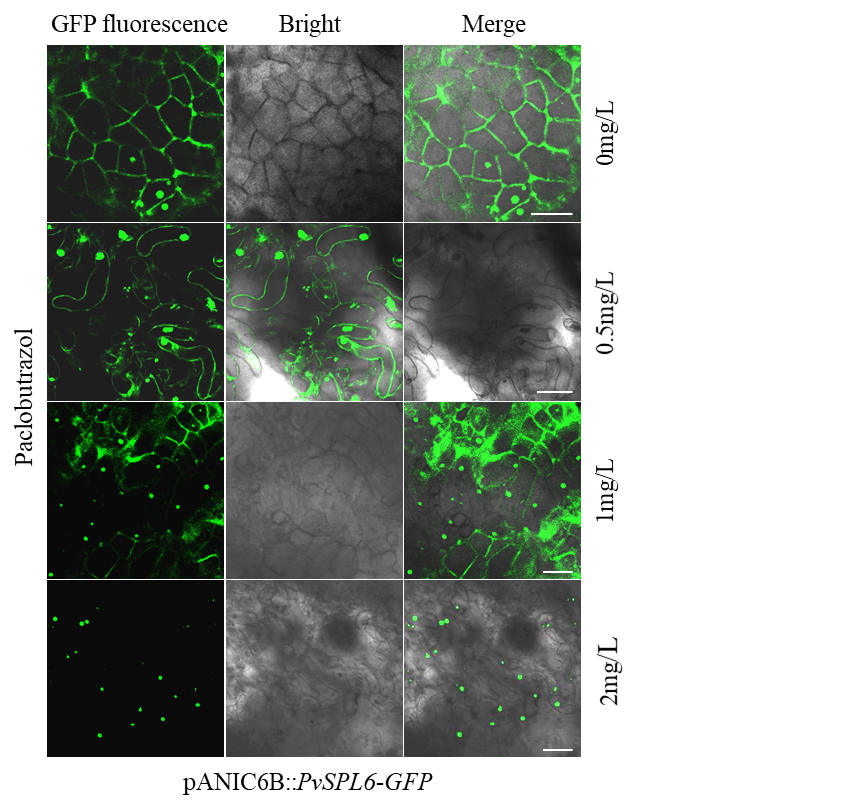


**Supplementary Figure 8.** **Subcellular localization analysis of differentiated callus from PvSPL6-GFP_OE_ transgenic plants in response to paclobutrazol.** Subcellular localization of PvSPL6 in response to different concentrations of paclobutrazol (0, 0.5, 1, and 2 mg/L). GFP fluorescence, green fluorescent signal; Bright, bright field signal; Merge, superimposed signal. Scale bar = 20 µm.

**Supplementary Figure 9**


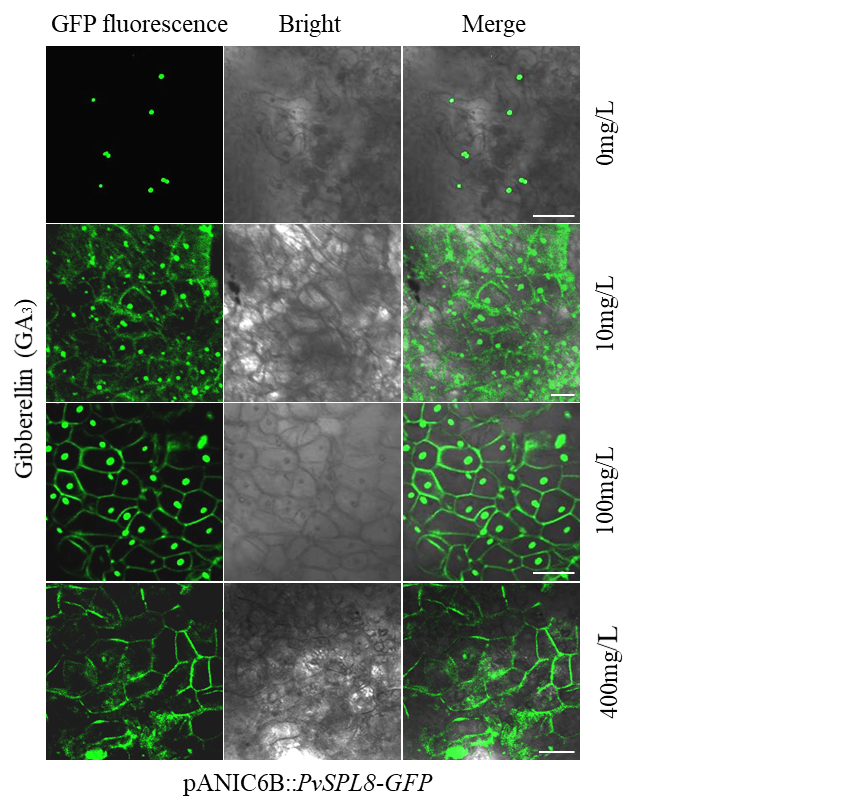


**Supplementary Figure 9** **Subcellular localization analysis of embryogenic callus from PvSPL8-GFP_OE_ transgenic plants in response to GA_3_.** Subcellular localization of PvSPL6 in response to different concentrations of GA_3_ (0, 10, 100, and 400 mg/L). GFP fluorescence, green fluorescent signal; Bright, bright field signal; Merge, superimposed signal. Scale bar = 20 µm.

**Supplementary Table 1 Sequences of the primers used in this study**

| **Expriment** | **Primer names** | **Primer sequences (5’-3’)** |
| --- | --- | --- |
| CDS cloning | *PvSPL6*-F | ATGAGAGCTAAGCAAGCTAGC |
|  | *PvSPL6*-R | TTATCTGATCTGGAAGTGGTTCCGT |
|  | *PvSPL6*-RNAi-F | CCCTTGCTTCGTGTCATCGT |
|  | *PvSPL6*-RNAi-R | TGCCGTAGCAGGGTTCTGTC |
| Plant vector construction | PGWC-*PvSPL6*-F | AAAGCAGGCTTTGACTTT  ATGAGAGCTAAGCAAGCTAGC |
|  | PGWC-*PvSPL6*-R | GCTGGGTCTAGAGACTT  TTATCTGATCTGGAAGTGGTTCCGT |
|  | PGWC-*PvSPL6*-RNAi-F | AAAGCAGGCTTTGACTTT  CCCTTGCTTCGTGTCATCGT |
|  | PGWC-*PvSPL6*-RNAi-R | GCTGGGTCTAGAGACTT  TGCCGTAGCAGGGTTCTGTC |
| Plant identification | *hph3* | AAGGAATCGGTCAATACACTACATGG |
|  | *hph4* | AAGACCAATGCGGAGCATATACG |
|  | *ZmUBQ-*F | TGTCGATGCTCACCCTGTTG |
|  | *Guslinker*-F | CGTCGTCGGTGAACAGGTAT |
|  | *Guslinker*-R | CACGCAAGTCCGCATCTTCA |
| Subcellular localization | 35S-*PvSPL6*-F | CGACTCTAGAAAGCTT  ATGAGAGCTAAGCAAGCTAGC |
|  | 35S-*PvSPL6*-R | CGGGCCCCTGCAGAAGC TCTGATCTGGAAGTGGTTCCGT |
|  | 35S-*PvSPL6*-N-F | CGACTCTAGAAAGCTT  ATGAGAGCTAAGCAAGCTAGC |
|  | 35S-*PvSPL6*-N-R | CGGGCCCCTGCAGAAGC  TTACTCCGCATGCGGCGACG |
|  | 35S-*PvSPL6*-C-F | CGACTCTAGAAAGCTT  GAGGACAGAACCCTGCTAC |
|  | 35S-*PvSPL6*-C-R | CGGGCCCCTGCAGAAGC TTATCTGATCTGGAAGTGGTT |
|  | 35S-*PvSPL2*-F | CGACTCTAGAAAGCTT  ATGAGTTCATTTGGGATGGACT |
|  | 35S-*PvSPL2*-R | CGGGCCCCTGCAGAAGC  GTGCATCAGGTCATAGTGGGA |
|  | PGWC-*PvSPL4*-F | AAAGCAGGCTTTGACTTT  ATGCACTCTAAGGCTCCACTTG |
|  | PGWC-*PvSPL4*-R | GCTGGGTCTAGAGACTT  GAGCGACCAGTCCGATGTGT |
|  | PGWC-*PvSPL6*-F | AAAGCAGGCTTTGACTTT  ATGAGAGCTAAGCAAGCTAGC |
|  | PGWC-*PvSPL6*-R | GCTGGGTCTAGAGACTT  TCTGATCTGGAAGTGGTTCCGT |
| qRT-PCR | *PvUBQ2*-qRT-F | TTCGTGGTGGCCAGTAAG |
|  | *PvUBQ2*-qRT-R | AGAGACCAGAAGACCCAGGTACAG |
|  | *PvSPL6*-qRT-F | CAGGTGATTAAGCAGGTACCCT |
|  | *PvSPL6*-qRT-R | GGCACAGGCGAACGAATTAC |
|  | *PvSPL7′*-qRT-F | CCAGCTCTCCTCCCCCGAC |
|  | *PvSPL7′*-qRT-R | TCTCGCAACGTGCAGGTCG |
|  | *PvSPL7*-qRT-F | ATTGCATTGCCCTACGTGCT |
|  | *PvSPL7*-qRT-R | ATAGCACATACACCTCCGGC |
|  | *PvSPL8*-qRT-F | AGCAAATTAACCAGCCATCG |
|  | *PvSPL8*-qRT-R | GAGCCCCTACGGGACTTGT |
|  | *PvSPL17*-qRT-F | GTGTGTGCGTGCGTCTACCCTACTA |
|  | *PvSPL17*-qRT-R | TTACAGGAGGTGATGGGAGCCG |
|  | *PvMADS32*-qRT-F | GACCTGCTGCTCCTCCTCTTCT |
|  | *PvMADS32*-qRT-R | GGATGTCCGTCCAGACCTTGGT |
|  | *PvMADS5*-qRT-F | CGGAACGGGCTGCTCAAGAA |
|  | *PvMADS5*-qRT-R | ATGCGGTATCGCTCCAATGTCT |

**Supplementary Table 2 Phenotypic statistics of PvSPL6-GFP_OE_ transgenic switchgrass plants**

| **Line** | **WT** | **PvSPL6-GFP_OE-1_** | **PvSPL6-GFP_OE-2_** | **PvSPL6-GFP_OE-3_** |
| --- | --- | --- | --- | --- |
| Expression levels of *PvSPL6* | 0.000243786623827227±5.49693311817553E-06 | 0.00341984314307325±0.000270107928446681 | 0.00529352330655955±0.000151272048201541 | 0.00488722032014908±0.00039896431535826 |
| Flowering time (day) | 128±1.58113883008419 | 108±2 | 96.8±1.48323969741913 | 100.2±1.48323969741913 |
| Plant height (cm) | 82.04±2.00698779268834 | 72.2±2.83284309484306 | 53.12±3.9467708319587 | 61.8±2.46069095987286 |
| Internode length (cm) | 9.46±0.618869937870632 | 7.46±0.343511280746353 | 5.66±0.304959013639538 | 6.36±0.288097205817759 |
| Number of internode | 6.4±0.547722558 | 5.4±0.547722558 | 4.4±0.547722558 | 5.2±0.447213595 |
| Number of tiller | 22.6±2.302172887 | 21.4±1.516575089 | 18.6±1.140175425 | 20.6±1.816590212 |
